# Methodological Evaluation of Micro-CT Analytical Parameters for Quantifying Bone Loss in a Murine Maxillary Peri-Implantitis Model

**DOI:** 10.3390/dj14060343

**Published:** 2026-06-05

**Authors:** Ofir Ginesin, Shiran Barsheshet-Karif, Yaniv Mayer, Eran Gabay, Yotam Bar-On, Hadar Zigdon-Giladi, Zvi Gutmacher

**Affiliations:** 1 The Ruth and Bruce Rappaport Faculty of Medicine, Technion—Israel Institute of Technology, Haifa 3525422, Israel; yaniv.mayer@technion.ac.il (Y.M.); eran.gabay@technion.ac.il (E.G.);; 2 Department of Periodontology, Rambam Health Care Campus, Haifa 3109601, Israel; 3 Laboratory for Bone Repair, Clinical Research Institute at Rambam (CRIR), Haifa 3109601, Israel; 4 Department of Prosthodontics, School of Graduate Dentistry, Rambam Health Care Campus, Haifa 3109601, Israel; 5 Department of Immunology, The Ruth and Bruce Rappaport Faculty of Medicine, Technion—Israel Institute of Technology, Haifa 3525422, Israel

**Keywords:** peri-implantitis, micro-CT, murine model, bone volume, volume of interest, threshold optimization, dental implant

## Abstract

**Background**: Peri-implantitis is a prevalent inflammatory condition leading to progressive bone loss around dental implants. In vivo studies widely use murine peri-implantitis models. Micro-computed tomography (micro-CT) is the gold standard method used to assess peri-implant bone changes in those models. However, no standardized protocol exists for image analysis, limiting comparability across studies. **Objective**: This study aimed to evaluate the effect of different micro-CT analysis parameters on bone measurements in a ligature-induced peri-implantitis mouse model. **Methods**: Seventeen C57BL/6J mice were divided into peri-implantitis (*n* = 8) and healthy (*n* = 9) groups. Following extraction and implant placement in the maxillary first molar region, peri-implantitis was induced via silk ligature placement. Micro-CT scans were analyzed by varying the volume of interest (VOI) size (2-thread vs. 5-thread spans), threshold values for bone and implant visualization, and measurement dimensionality (2D linear vs. 3D volumetric). Statistical significance (*p* < 0.05) was determined using unpaired *t*-tests or Mann–Whitney U tests, following Shapiro–Wilk normality assessment. **Results**: The 2-thread VOI yielded a highly significant difference in bone volume fraction (BV/TV) between groups (68.86 ± 4.97% vs. 50 ± 4.32%, *p* < 0.0001, Cohen’s d = 4.05), while the 5-thread VOI showed no significant difference. Thresholds of 60 and 200 were utilized for bone and implant segmentation, respectively, selected based on high inter-examiner reliability (Cohen’s kappa = 0.844 and 0.825, respectively). Two-dimensional analysis confirmed greater mesio-distal loss in the peri-implantitis group. **Conclusions**: A 2-thread coronal VOI combined with optimized thresholds provides a promising analytical approach for evaluating localized peri-implant bone changes in this exploratory model. While this approach demonstrated pronounced responsiveness within our dataset, further external validation across different imaging systems and operators is required to establish its role as a universal standard.

## 1. Introduction

Dental implants are a widely used treatment for replacing missing teeth. However, they are susceptible to biological complications, most notably peri-implantitis. According to the 2017 World Workshop on the Classification of Periodontal and Peri-Implant Diseases and Conditions, peri-implantitis is defined as a pathological condition occurring in tissues around dental implants, characterized by inflammation of the peri-implant mucosa and progressive loss of supporting bone [1]. Recent flow cytometry analysis of human peri-implantitis lesions has further characterized the immune cell infiltrate, identifying specific innate and adaptive immune cell populations involved in the inflammatory process [2]. The condition affects approximately 25% of implant patients, and the incidence over a 5–23-year follow-up period is estimated at 28–56% [3,4]. Clinically, peri-implantitis is characterized by mucosal swelling, suppuration, and progressive destruction of the bone surrounding the implant, thereby compromising implant longevity [4].

Current treatments for peri-implantitis include non-surgical and surgical approaches. However, the success of available treatments remains unpredictable, and clinical outcomes are often less favorable than expected [5].

Ethical constraints preclude the induction of peri-implantitis in humans for research purposes. Therefore, animal models are essential for studying the underlying mechanisms and developing effective treatments. Peri-implantitis has been successfully modeled in several animal species, including dogs, rabbits, and mice [6,7]. Among these, mice offer particular advantages, including the availability of genetically modified strains, relatively low cost, and short breeding cycles, making them an ideal model for peri-implantitis research, particularly for elucidating the underlying pathogenesis of the disease [7].

The most commonly used method for inducing peri-implantitis in mice involves the placement of a ligature around the implant [8,9]. Ligatures promote local bacterial accumulation, enhancing bacteria-driven inflammation and subsequent bone resorption. Part of the associated bone loss may also be attributed to mechanical friction between the ligature and the surrounding tissues. This model enables controlled disease initiation at a defined time point and is both reproducible and cost-effective [10]. Alternatively, peri-implantitis can be induced through bacterial infection or lipopolysaccharide (LPS) injection [11], both of which produce predictable peri-implant bone loss. Nguyen Vo et al. [8] investigated peri-implantitis induction using localized LPS injections and ligature placement, reporting comparable levels of peri-implant bone loss at 8 and 12 weeks, respectively.

Assessment of peri-implantitis in murine models involves a combination of clinical evaluation, imaging, histological, and molecular methods. Bone loss is the primary outcome measure, and micro-computed tomography (micro-CT) has become the preferred imaging modality for quantifying peri-implant bone changes. Compared with histomorphometry, micro-CT offers several important advantages: it provides three-dimensional volumetric data, reduces measurement errors during processing and analysis, yields results more rapidly, and preserves the specimen allowing repeated measurements of the same sample or additional biological or mechanical testing [12,13,14,15,16].

Micro-CT is widely utilized in rodent dentoalveolar research [17]. While established protocols exist for periodontitis analysis [18,19] and general guidelines have been proposed [9], a standardized protocol for micro-CT data analysis in murine peri-implantitis has yet to be established [20]. Studies vary considerably in key analytical parameters. For example, the selection of the volume of interest (VOI) varies significantly between studies; while some protocols quantify residual bone support, others measure the bone loss [20]. In the absence of evidence-based guidance, researchers frequently utilize broad VOIs that encompass apical bone regions unaffected by early-stage disease. This inclusion potentially dilutes the inflammatory biological signal, thereby compromising the statistical power and sensitivity of in vivo experiments. This methodological fragmentation was highlighted in a systematic review by Chew et al., where the lack of standardized VOI definitions precluded a formal meta-analysis [21], further emphasizing the need for a unified analytical framework. Inconsistencies in other critical analytical parameters, such as the thresholds utilized for bone and implant segmentation, are also common. Studies may omit specific threshold values [22] or utilize manual, specimen-specific selection [23]. This lack of methodological transparency not only hinders the direct comparison of results across different imaging protocols but also compromises the ethical goal of animal reduction by preventing accurate, evidence-based sample size calculations. Therefore, the primary aim of this study was to examine the effect of different micro-CT analysis parameters on bone measurements in a murine peri-implantitis model. By quantitatively assessing the sensitivity and reproducibility of different workflows, this research seeks to address current methodological gaps and to provide a framework for maximizing the signal-to-noise ratio in peri-implant research. A secondary aim was to propose a reproducible analytical framework for micro-CT image analysis that can be adopted by future studies to facilitate reliable and comparable bone assessments.

## 2. Materials and Methods

### 2.1. Ethics Statement

All procedures were conducted in accordance with protocols approved by the Technion Committee on Animal Research (approval number: IL-092-05-2023, Issued date 29 May 2023) and adhered to the ARRIVE guidelines [24] and relevant regulations.

### 2.2. Animals and Housing

Six-week-old male C57BL/6J mice (22–24 g; ENVIGO, Ness Ziona, Israel) were used in this study. Animals were housed in ventilated cages in a temperature-controlled environment with ad libitum access to food and water under a 16 h light/8 h dark cycle.

### 2.3. Experimental Design and Surgical Procedures

Seventeen mice were divided into two groups: a control (healthy) group (*n* = 9) and an experimental (peri-implantitis) group (*n* = 8). Group allocation was performed by randomly assigning entire cages to either the healthy or the peri-implantitis group to ensure that all animals within a single cage received the same treatment protocol. Cage-level group allocation was predefined and finalized prior to the initial surgical procedure (extraction). All surgical procedures were performed under a surgical microscope (Zeiss, Oberkochen, Germany). Mice were anesthetized via intraperitoneal injection of ketamine (80 mg/kg) and xylazine (16 mg/kg), supplemented with local infiltration of 2% lidocaine with epinephrine (1:100,000). For analgesia, long-acting buprenorphine (0.3 mg/kg) was administered subcutaneously during each surgical procedure.

**Figure 1:**
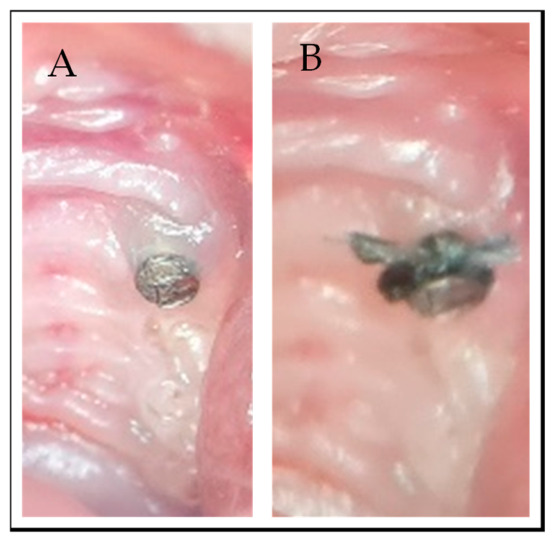
Induction of peri-implantitis in a mouse model: (**A**) 3 weeks post extraction; miniature dental implant is placed (**B**) after 3 weeks—silk ligature is tied around the dental implant.

The experimental timeline was as follows: at week 0, the maxillary left first molar was extracted. Three weeks later (week 3), a titanium para-post implant (0.6 mm diameter, titanium-6 aluminum-4 vanadium alloy, “Retopins,” Fairfax Medical Products, London, UK) was inserted into the extraction site. These micro-implants feature a parallel-walled macro-design with defined, uniform threads and a machined surface. After an additional three weeks of healing (week 6), a 5-0 silk ligature was tied around the supragingival portion of the implant to induce peri-implantitis in the experimental group [10] (Figure 1B). Two weeks following ligature placement (week 8), the animals were sacrificed and the maxillae were harvested. Specimens were fixed in formalin for 24 h and subsequently stored in 70% ethanol at room temperature until micro-CT scanning.

### 2.4. Micro-CT Scanning

Specimens were positioned with the implant perpendicular to the scanner tube, and all samples were oriented as consistently as possible. Micro-CT scans were acquired using a SkyScan 1276 scanner (Bruker, Kontich, Belgium) under the following strictly identical acquisition parameters: source voltage 70 kV, source current 60 µA, pixel size 10.5 µm, exposure time 3000 ms, rotation step 0.3°, and frame averaging ON.

### 2.5. Data Analysis

Volumetric data were reconstructed using NRecon software (Version 1.7.5.4, Bruker). The beam hardening correction was set to 0, smoothing was set to 0, and a ring artifact correction of 8 was applied. DataViewer software (Version 1.5.6.2, Bruker) was used to orient each sample so that the bony ridge was parallel to the X-axis, regardless of the implant angle. The same volume of interest (VOI) was defined for all samples.

**Figure 2:**
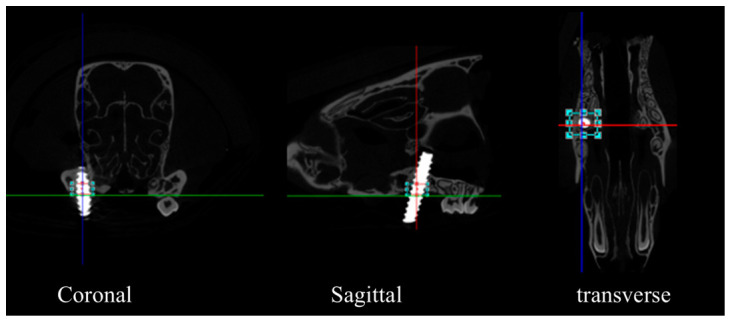
Representative micro-CT images and coordinate alignment in DataViewer software. To ensure high-precision measurements regardless of the physical implant tilt, each sample was oriented so that the bony ridge remained parallel to the horizontal X-axis. Blue square marks the volume of interest in the different sections: coronal, sagittal, and transverse.

To eliminate operator variance and subjective bias, the entire downstream analysis was performed by the same single examiner who remained fully blinded to the experimental groups and sample origins throughout. The same standardized volume of interest (VOI) was defined for all samples. Two peri-implant analysis heights were predefined using the implant thread count as an anatomical reference: the coronal 2-thread span and the 5-thread span. Consistency in VOI positioning was achieved by using the alveolar crest as the primary vertical reference point. For each sample, the reference level was defined by a line extending between the most coronal points of the proximal bony crest on both sides of the implant (mesial–distal for sagittal views, buccal–lingual for coronal views) (Figure 2). From this crest-based reference, the 2-thread VOI extended apically to the inferior border of the second thread, and the 5-thread VOI to the inferior border of the fifth thread, both aligned along the implant long axis. The 2-thread VOI corresponded to a sagittal height of approximately 860 µm (voxel size of 10.5 µm per slice over 85 slices), and the 5-thread VOI corresponded to approximately 2310 µm (10.5 µm per slice over 220 slices). All micro-CT metrics were computed using CTAn software (Version 1.18.8.0, SkyScan, Bruker) in both volumes to determine which yielded superior performance. The primary VOI was designated as the one demonstrating better repeatability and consistent biological separation between groups.

### 2.6. Two-Dimensional Measurements

**Figure 3:**
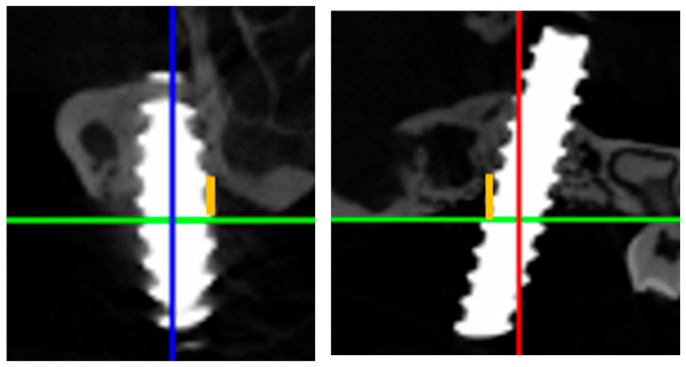
Example of two-dimensional linear measurement. Green line—bone peak, orange line—the distance from bone peak to the level of bone near the implant. By analyzing both mesio-distal and bucco-lingual aspects, the full extent of the vertical bone shelf reduction is captured.

Linear measurements of bone loss in the mesio-distal and bucco-lingual aspects were performed using DataViewer software on selected coronal and sagittal sections. A reference line was first drawn connecting the highest bony peaks adjacent to the implant site. The extent of bone loss was then defined as the perpendicular distance from this reference line to the deepest point of the alveolar bone crest (Figure 3). Bone loss values were recorded in micrometers (µm).

### 2.7. Three-Dimensional Measurements

The initial bone threshold was adapted from the methodology of Carli et al. (2012), which employed a threshold of 40% of the maximal grayscale value (80/200) to segment bone from non-bone tissue [25].

**Figure 4:**
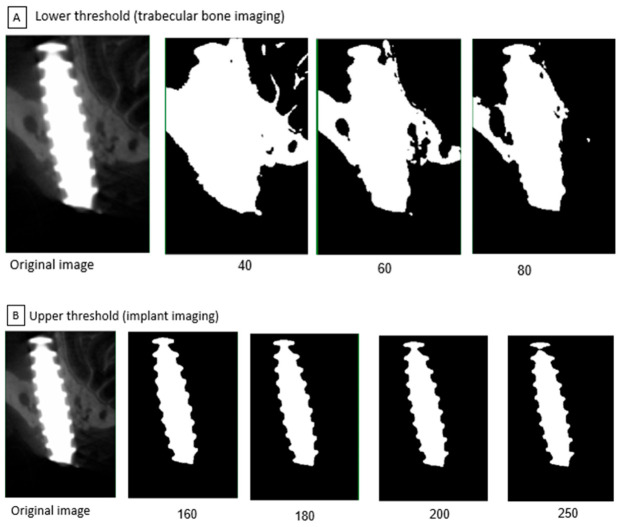
Visual calibration of different segmentation thresholds. To ensure the most accurate digital reconstruction, various settings were evaluated to find the ideal balance for bone and implant visualization. (**A**) Lower threshold example—left, original picture and 3 pictures of different thresholds (40, 60, 80). (**B**) Upper threshold example—left, original picture and 3 pictures of different thresholds.

This thresholding strategy was further refined based on the distinct physical properties of the materials involved. Titanium possesses a significantly higher electron density than the hydroxyapatite in bone, resulting in two distinct peaks in the grayscale histogram. By isolating these peaks, we ensured that the volume of interest (VOI) accurately represented the biological interface while minimizing artifacts such as metallic blooming and partial volume effects (Figure 4A,B).

To determine the optimal segmentation thresholds, two independent observers (O.G. and S.B.K.) evaluated five samples from each group. For each sample, a representative section was displayed at multiple thresholds and compared with the original image. Lower thresholds of 40, 60, and 80 were tested for bone visualization, and upper thresholds of 160, 180, 200, and 250 were tested for implant visualization. Each observer independently identified the optimal lower threshold for bone and upper threshold for implant visualization. Based on the consensus between the two examiners and the degree of inter-examiner reliability, fixed threshold values were established for both bone and the implant. Following this calibration, all samples were analyzed in a randomized sequence using CTAn software (Bruker, Belgium), with group allocations finalized prior to the initial procedures.

### 2.8. Sample Size Calculation

Sample size was determined by a priori power analysis based on data from a previous murine peri-implantitis study [8]. The calculation was based on an expected mean bone volume fraction of 0.7 ± 0.07 for the healthy group and 0.6 for the peri-implantitis group at the 2-week time point yielding a Cohen’s d effect size of 1.43. With the probability of a Type I error set at 0.05 and the statistical power set at 0.80, a minimum sample size of 8 animals per group was indicated to detect significant differences between the groups.

### 2.9. Statistical Analysis

The individual animal was defined as the statistical unit (n) for all metric evaluations and comparative analyses. Statistical parameters including means, medians, ranges, standard deviations (SD), and standard errors of the mean (SEM) were calculated. Data were first screened for outliers using the ROUT method (Q = 2%), and the Shapiro–Wilk test was employed to assess the normality of data distribution. Inter-examiner reliability was assessed using a representative subset of 10 cases. Two independent, blinded examiners evaluated both the bone and implant thresholds. Reliability was quantified using Cohen’s kappa. Additionally, intra-examiner consistency was evaluated on a confirmatory subset. For comparisons between groups, an unpaired Student’s *t*-test was used for normally distributed data, while the Mann–Whitney U test was applied for non-parametric data. To determine the magnitude of the differences between groups, effect sizes were calculated using Cohen’s d, with calculations performed using Microsoft Office 365 (Microsoft Corp., Redmond, WA, USA). The significance level of *p* < 0.05 was set. Statistical analysis was conducted using GraphPad Prism 9.0 (GraphPad Software, Inc., San Diego, CA, USA).

## 3. Results

### 3.1. Two-Dimensional Bone Loss

**Figure 5:**
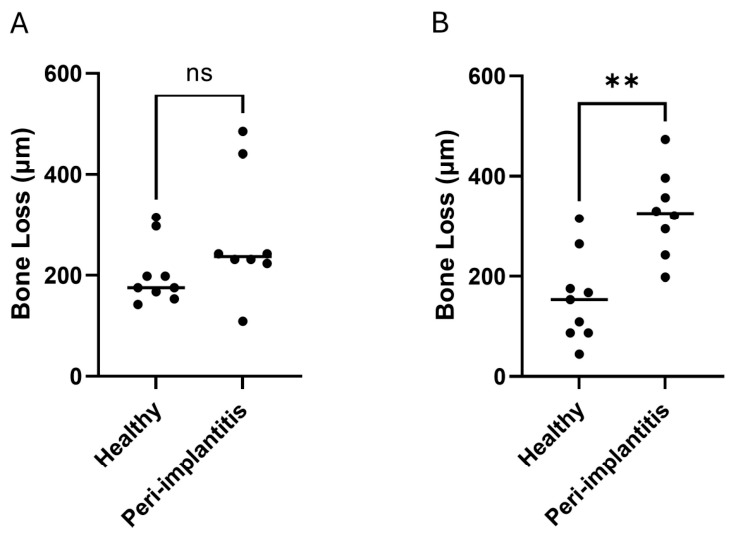
Dot plot of two-dimensional bone loss measurements in (**A**) sagittal and (**B**) coronal cross-section of healthy and peri-implantitis sites. Each dot represents one sample. *n* = 9 for the healthy group; *n* = 8 for the peri-implantitis group. **—*p*-value < 0.01; ns—not significant.

Vertical two-dimensional bone loss measurements of healthy and peri-implantitis samples are presented in Table 1 and Figure 5. Two-dimensional sagittal linear measurements were found to follow a normal distribution and were analyzed using Student’s *t*-test. In contrast, the 2D coronal linear measurements exhibited a non-normal distribution, requiring the use of the non-parametric Mann–Whitney U test. In the sagittal (mesio-distal) plane, the peri-implantitis group demonstrated significantly greater bone loss (95% CI: 79.99 to 259.8, *p*-value = 0.001, Cohen’s d = 1.95) (Figure 5A), with values ranging from 198 to 472 µm (mean: 326 ± 89 µm) compared to the bone loss in the healthy group, which ranged from 44 to 315 µm (mean: 156 ± 85 µm).

Coronal (bucco-lingual) measurements followed a similar upward trend in the disease group (110 to 484 µm mean: 275 ± 119 µm) compared to bone loss ranging from 143 to 315 µm (mean: 203 ± 57 µm) in the healthy group. The difference between the groups did not reach statistical significance (*p*-value = 0.106, Cohen’s d = 0.79) (Figure 5B).

### 3.2. Three-Dimensional Bone Volume

The inter-examiner reliability analysis demonstrated high consistency for both analytical parameters. For the bone threshold, the examiners reached agreement in 9 out of 10 cases, yielding a 90% agreement and a Cohen’s kappa of 0.844. Similarly, for the implant threshold, the examiners achieved 91.7% agreement with a Cohen’s kappa value of 0.825. Cohen’s kappa values above 0.80 are generally interpreted as indicating almost perfect agreement according to established classification systems [26]. These values confirm that the visual calibration process effectively ensured high consistency and minimized observer subjectivity. The intra-examiner re-evaluation resulted in full agreement for all parameters. Threshold optimization revealed that a lower threshold of 60 best demonstrated trabecular bone structure, while an upper threshold of 200 optimally delineated the implant (Figure 4). Bone analysis was subsequently performed using these threshold settings.

A preliminary analysis was performed to determine the optimal VOI size for peri-implant micro-CT measurements. Quantitative data for the two predefined VOIs are presented in Table 2. For the 2-thread VOI, two outliers were removed in the healthy group (samples 1, 5) and one outlier in the peri-implantitis group (sample 6). Following the exclusion of these outliers, the Shapiro–Wilk test confirmed that all 3D volumetric datasets in both the healthy and peri-implantitis groups followed a normal distribution.

**Figure 6:**
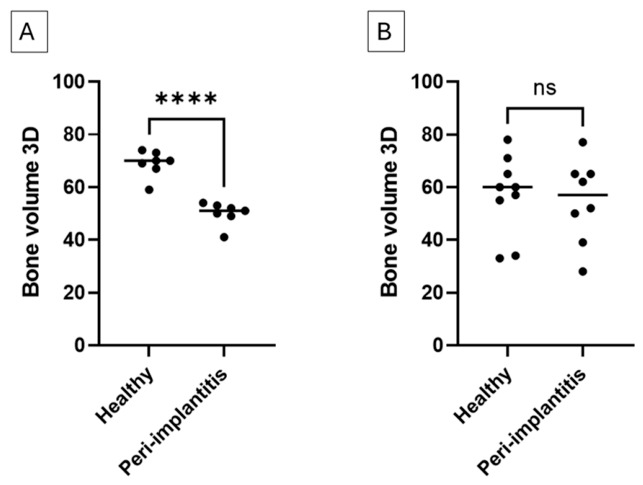
Dot plot of bone volume out of total volume (BV/TV %) in two different volumes of interest (VOI) (**A**) for 2-thread (following outlier removal *n* = 7 per group); (**B**) for 5-thread (*n* = 9 for healthy, *n* = 8 for peri-implantitis). ****—*p*-value < 0.0001, ns—not significant.

The mean bone volume fraction (BV/TV) was 68.86 ± 4.97% in the healthy group and 50 ± 4.32% in the peri-implantitis group, with a highly significant difference between groups (95% CI: −24.27% to −13.45%, *p* < 0.0001, Cohen’s d = 4.05) (Figure 6A). To confirm the robustness of our conclusions, a sensitivity analysis was performed. When all three outliers were included in the analysis, the data exhibited a non-normal distribution. Under these conditions, a non-parametric Mann–Whitney U test still revealed a statistically significant difference between the groups (*p* = 0.016).

In contrast, for the 5-thread VOI, BV/TV values were 57.00 ± 15.12% in the healthy group and 54.75 ± 15.82% in the peri-implantitis group, showing no statistically significant difference (95% CI: −18.25 to 13.75, *p* = 0.768, Cohen’s d = 0.15) (Figure 6B). Based on these results, the 2-thread VOI was selected as the primary analysis volume for further analysis.

## 4. Discussion

Peri-implantitis is a common inflammatory condition that leads to progressive bone loss around dental implants, posing a significant clinical challenge. Animal models, particularly mice, are widely used to study the disease under controlled conditions. In murine studies, peri-implantitis is frequently induced by ligature placement, and bone changes are commonly evaluated using micro-CT. While micro-CT provides high-resolution three-dimensional imaging for quantifying bone loss, its analysis requires specialized image processing with multiple adjustable parameters.

A review of the literature reveals a lack of standardized protocols for micro-CT image analysis in murine peri-implantitis research, with studies adopting varying methods for VOI selection, threshold determination, and measurement dimensionality [10,23,27,28]. This concern was recently highlighted in comprehensive reviews [20,21] which emphasize that without a uniform consensus on volume of interest (VOI) selection and thresholding, the internal validity of different bone loss models is difficult to verify.

The existing literature reveals two fundamentally different philosophical approaches to quantification that our study seeks to reconcile. For instance, Tzach-Nahman et al. evaluated Residual Supportive Bone Volume (RSBV) by setting an occlusal reference plane and a parallel apical plane strictly 800 µm apical to the first implant thread, specifically analyzing the buccal bone plate [27]. While this provides a snapshot of the remaining structure, the inherent complexity of mouse anatomy and the limited vertical height of the murine alveolar ridge present significant challenges for such protocols. Inconsistency may stem from anatomical features such as the sinus cavity, which can interfere with and skew the results by including bone or air spaces biologically unrelated to the peri-implant process. Consequently, the current study advocates for the direct measurement of bone loss within a localized VOI rather than the indirect assessment of residual bone.

In contrast, Hiyari et al. focused on quantifying bone loss by normalizing measurements starting 10 slices below the junction of the implant head and shaft to account for the biologic soft tissue seal [22]. However, this normalization strategy presents a potential risk, as it may fail to capture significant bone loss occurring within that specific coronal zone. Rather than the generalized thresholds or offsets proposed, we suggest using individualized internal markers. By anchoring the VOI to the adjacent bony ridges and using a fixed thread-based coordinate system, our protocol is designed to optimize the capture of the active resorption zone thereby minimizing the loss of coronal data or over-extending into apical anatomical noise. Consequently, this proposed coordinate framework may streamline replication efforts and support better data alignment across similar research settings.

A significant barrier to the reproducibility of micro-CT data is the subjectivity inherent in threshold selection. The initial bone threshold was adapted from the methodology of Carli et al. (2012), who employed a baseline threshold of 40% of the maximal grayscale value (corresponding to threshold of 80) to segment bone from non-bone tissue [25]. However, to better account for the specific imaging characteristics of our model, this strategy was further refined through a consensus evaluation by two independent observers, ultimately setting the bone threshold at 60.

Because titanium possesses a significantly higher electron density than the hydroxyapatite found in bone, the grayscale histogram exhibited two distinct peaks. This allowed for the identification of a suitable range to isolate the fixture from the surrounding tissue, leading to the selection of 200 as the implant threshold. By utilizing these dual thresholds, we successfully isolated the biological interface while minimizing artifacts such as metallic blooming and partial volume effects.

While these parameters were optimized for our specific hardware and acquisition settings, we acknowledge that threshold selection involves an inherent element of subjectivity. These values should be considered as a baseline and should be further verified by other research groups utilizing different micro-CT systems and scanning parameters. Standardizing the reporting of these values, even when subjectively determined, is a critical step toward creating transparent and comparable datasets required for murine peri-implantitis research.

Comparison of VOIs encompassing two versus five implant threads revealed that differences between the healthy and peri-implantitis groups were more pronounced when analysis was restricted to the coronal two threads (*p*-value < 0.0001), whereas the five-thread VOI did not reach statistical significance (*p* > 0.05). These findings suggest that a smaller peri-implant VOI enhances the sensitivity of micro-CT to detect disease-associated bone changes, likely by minimizing variability from more apical regions less affected by early bone loss. However, restricting analysis to a smaller region carries the potential risk of missing changes that extend more apically. Considering this balance, our results suggest that for a 2-week murine peri-implantitis model, a VOI extending two threads apically from the alveolar crest provides an optimized balance between pronounced responsiveness and biological relevance. This localized approach effectively captures the primary zone of inflammatory resorption while minimizing the ‘dilution effect’ from stable apical bone. Our data support this as a promising analytical framework. However, it has only been examined specifically for this murine strain, implant macro-design, peri-implantitis induction method, and micro-CT analysis settings. Consequently, in studies involving more protracted time points or different implant geometries, the VOI may need to be adjusted to reflect the potentially more extensive apical progression of the disease.

Beyond its technical advantages, the standardization of micro-CT analysis protocols serves a critical ethical purpose by aligning with the 3Rs principles (Replacement, Reduction, and Refinement). The superior sensitivity of the 2-thread protocol is quantitatively confirmed by the massive disparity in effect sizes observed between the two volumes of interest. While the 5-thread VOI yielded a negligible effect size (d = 0.15), indicating that the biological signal was heavily diluted by unaffected apical bone, the 2-thread VOI yielded a robust effect size (d = 4.05). This demonstrates that the 2-thread VOI effectively isolates the specific region where bone resorption is most active. By maximizing the detected difference between healthy and peri-implantitis groups, this refined approach directly improves the efficiency of power analyses for in vivo studies. Because biological separation is more pronounced, researchers can achieve statistically significant results with a smaller sample size, thereby effectively reducing the number of animals required for research. Adopting such a sensitive and systematic analytical strategy may help future studies generate more comparable data, while maintaining a steadfast commitment to ethical animal welfare. However, further validation in different experimental settings is warranted to confirm its broad generalizability.

Statistically significant differences in bone loss between the peri-implantitis and healthy groups were evident in both two-dimensional and three-dimensional analyses. Our two-dimensional findings are consistent with those of Pirih et al. [10], who reported mean distances from the implant head to the bone crest of 0.422 ± 0.019 mm in the experimental group and 0.226 ± 0.016 mm in the healthy group. In our study, comparable values were observed: 0.326 mm in the peri-implantitis group and 0.156 mm in the healthy group.

In the current study, 2D sagittal linear measurements demonstrated a statistically significant difference between the peri-implantitis and the healthy groups, while measurements in the coronal dimension did not reach significance. This disparity is not unique to our findings but reflects a broader methodological challenge in the literature. While some researchers average measurements from four aspects [22,29,30,31], others have reported only mesial and distal bone levels in micro-CT assessments [8], often deferring buccal and palatal analysis to histomorphometry.

The lack of significance in the coronal plane may be further explained by the fact that these measurements were not normally distributed, unlike the sagittal data. This likely stems from the inherent anatomical asymmetry of the murine maxilla. While we expect relatively similar bone morphology at the mesial and distal interfaces (sagittal plane), the coronal plane is characterized by a contrast between the palatal and the buccal bone.

Inconsistencies in threshold selection across studies further contribute to difficulties in comparing results. Varon-Shahar et al. [23] noted that threshold values were selected manually without reporting the specific values used. Other studies employed automated threshold selection tools provided by imaging software. This variation in threshold-setting methods underscores the need for a standardized approach, as proposed in the present study. Analysis of the images in the current study revealed that the ideal threshold of 60 is optimal for visualizing peri-implant bone, as it minimizes scattering while maximizing bone clarity. For implant visualization, a threshold of 200 is recommended. These values are essential for ensuring consistent and comparable analysis.

## 5. Limitations

This study has several limitations. First, a single micro-CT device was used. Consequently, variations across different scanners may necessitate protocol adjustments. Second, the specific implant used, both its dimensions and material, may have influenced biological responses and imaging outcomes. Consequently, our proposed VOI and threshold segmentation parameters may not be directly transferable to implants with alternative diameters, thread geometries, alloy compositions, or surface treatments. Third, specimen preparation was performed by a single clinician, and inter-operator variability was not assessed. Fourth, the study examined a single time point (2 weeks post-ligature placement); the optimal VOI may differ for longer disease durations where bone loss extends more apically. Fifth, only one mouse strain (C57BL/6J) and one induction method (ligature) were tested, and generalizability to other strains or induction methods requires further validation. Sixth, because group allocation occurred at the cage level, individual animals shared a microenvironment and may not represent completely independent statistical units. Seventh, the exclusion of multiple outliers from a relatively small dataset must be interpreted with caution. These outliers may represent minor biological or healing variations at the extraction site prior to implant placement, which altered subsequent peri-implant bone responses. However, our comprehensive sensitivity analysis demonstrates that the direction and statistical significance of the group separation remain robust regardless of outlier handling. Finally, the sample size, although based on power analysis, was relatively small. Despite these limitations, the proposed protocol may serve as a useful tool for improving the consistency of micro-CT data analysis in murine peri-implantitis research.

## 6. Conclusions

This study provides preliminary evidence that micro-CT analytical parameters, specifically the selection of the volume of interest (VOI), significantly influence the detection of bone loss in a murine ligature model. Our findings suggest that a localized, coronal-centric (2-thread) VOI showed greater group separation in this dataset within this specific experimental context, yielding a significantly higher effect size than more global assessments.

While these results highlight the potential of a thread-centric coordinate system to serve as a proposed framework for direct bone loss quantification, we emphasize that these conclusions are context-dependent. The broad adoption of such a protocol requires further external validation across diverse micro-CT systems, various implant designs, different operators, varied experimental settings, and different disease durations to ensure international comparability. Furthermore, the pronounced responsiveness of this proposed framework provides a statistical basis that may facilitate a reduction in the number of animals required for future in vivo studies, aligning with the 3Rs principle. Ultimately, this work serves as a starting point for improving the transparency and reproducibility of micro-CT reporting in the field of peri-implantitis.

## Figures and Tables

**Table 1:** Two-dimensional peri-implant bone loss (µm) measured in sagittal (mesio-distal) and coronal (bucco-lingual) sections.

Group	Sample	Sagittal Section—MD (µm)	Coronal Section—BL (µm)
**Peri-implantitis**	1	472	231
	2	294	242
	3	357	231
	4	242	110
	5	330	484
	6	396	440
	7	198	242
	8	321	223
		Mean: 326 ± 89	Mean: 275 ± 119
**Healthy**	1	315	315
	2	88	198
	3	168	168
	4	176	143
	5	154	297
	6	44	176
	7	110	154
	8	88	176
	9	264	198
		Mean: 156 ± 85	Mean: 203 ± 57

**Table 2:** Bone volume fraction out of the total volume (BV/TV, %) measured in 2-thread and 5-thread volumes of interest. Values marked with an asterisk (*) were identified as outliers using the ROUT method (Q = 2%) and were excluded from the final statistical analysis and mean calculations.

Group	Sample	2-Thread BV/TV (%)	5-Thread BV/TV (%)
**Peri-implantitis**	1	50	77
	2	53	50
	3	51	65
	4	54	62
	5	41	39
	6	23 *	28
	7	49	65
	8	52	52
	Mean ± SD (with outliers)	46.63 ± 10.35 (*n* = 8)	54.75 ± 15.82 (*n* = 8)
	Mean ± SD (without outliers)	50.00 ± 4.32 (*n* = 7)	54.75 ± 15.82 (*n* = 8)
**Healthy**	1	36 *	34
	2	69	57
	3	67	65
	4	70	71
	5	36 *	33
	6	73	60
	7	59	55
	8	70	60
	9	74	78
	Mean ± SD (with outliers)	61.56 ± 15.11 (*n* = 9)	57.00 ± 15.12 (*n* = 9)
	Mean ± SD (without outliers)	68.86 ± 4.97 (*n* = 7)	57.00 ± 15.12 (*n* = 9)

## Data Availability

The datasets generated and analyzed during the current study are available from the corresponding author upon reasonable request.
